# SMARCA4-Deficient Undifferentiated Tumor Presenting as a Tonsillar Mass With Widespread Metastases: A Rare Head and Neck Manifestation and Complete Response to Chemoimmunotherapy

**DOI:** 10.7759/cureus.90665

**Published:** 2025-08-21

**Authors:** Julie Huynh, Joshua Segal, Lauren Eisenbud, Rodolfo Gutierrez, Samuel J Slomowitz

**Affiliations:** 1 Hematology and Medical Oncology, University of California, Los Angeles, Los Angeles, USA; 2 Pathology, TOPA Diagnostics, Los Angeles, USA

**Keywords:** cancer immunotherapy, pembrolizumab, smarca4 deficient, smarca4-deficient undifferentiated tumor, thoracic smarca4-deficient undifferentiated tumor

## Abstract

SWI/SNF-related, matrix-associated, actin-dependent regulator of chromatin, subfamily A (SMARCA4)-deficient undifferentiated tumors (SMARCA4-UTs) are rare, aggressive malignancies typically arising in the thoracic cavity. We present a case of a 73-year-old male who initially presented with symptoms consistent with acute tonsillitis but rapidly developed odynophagia, dysphagia, cervical lymphadenopathy, and profound weight loss. Imaging revealed a left tonsillar mass with extensive metastases to lymph nodes, lungs, bones, and mediastinum. A biopsy of the tonsillar mass confirmed a SMARCA4-deficient undifferentiated tumor. The patient underwent systemic treatment with carboplatin, paclitaxel, and pembrolizumab, complicated by refeeding syndrome, perforated appendicitis, and pulmonary embolism. Despite this, he achieved complete remission after six cycles and continues on maintenance pembrolizumab. This case illustrates the potential for durable response with chemoimmunotherapy in this rare and aggressive malignancy, even when presenting in atypical anatomic locations.

## Introduction

SWI/SNF-related, matrix-associated, actin-dependent regulator of chromatin, subfamily A (SMARCA4)-deficient undifferentiated tumors are high-grade neoplasms defined by the loss of SMARCA4, also known as Brahma-related gene 1 (BRG1), a key catalytic subunit of the SWI/SNF chromatin remodeling complex [1]. These tumors most frequently arise in the thoracic cavity, particularly in the mediastinum and lungs, and are associated with a poor prognosis. Extrapulmonary manifestations, especially in the head and neck region, are exceedingly rare, with only one case reported in the literature [2]. Because of the rarity and aggressive behavior, diagnosis and management are often challenging. Here, we describe a rare case of a SMARCA4-deficient tumor originating in the tonsil with widespread metastasis at presentation and a notable complete response to chemoimmunotherapy.

## Case presentation

A 73-year-old male with a history of smoking, type 2 diabetes, and hypertension presented to urgent care with a sore throat. He was initially treated for presumed acute tonsillitis despite the lack of fever or leukocytosis. However, over the next two weeks, the patient developed progressive odynophagia, dysphagia, and visibly enlarging left tonsillar and cervical neck masses. He experienced a rapid 30-pound weight loss and became severely deconditioned. On exam, the left tonsil was enlarged, malodorous, and free from the soft palate.

**Figure 1:**
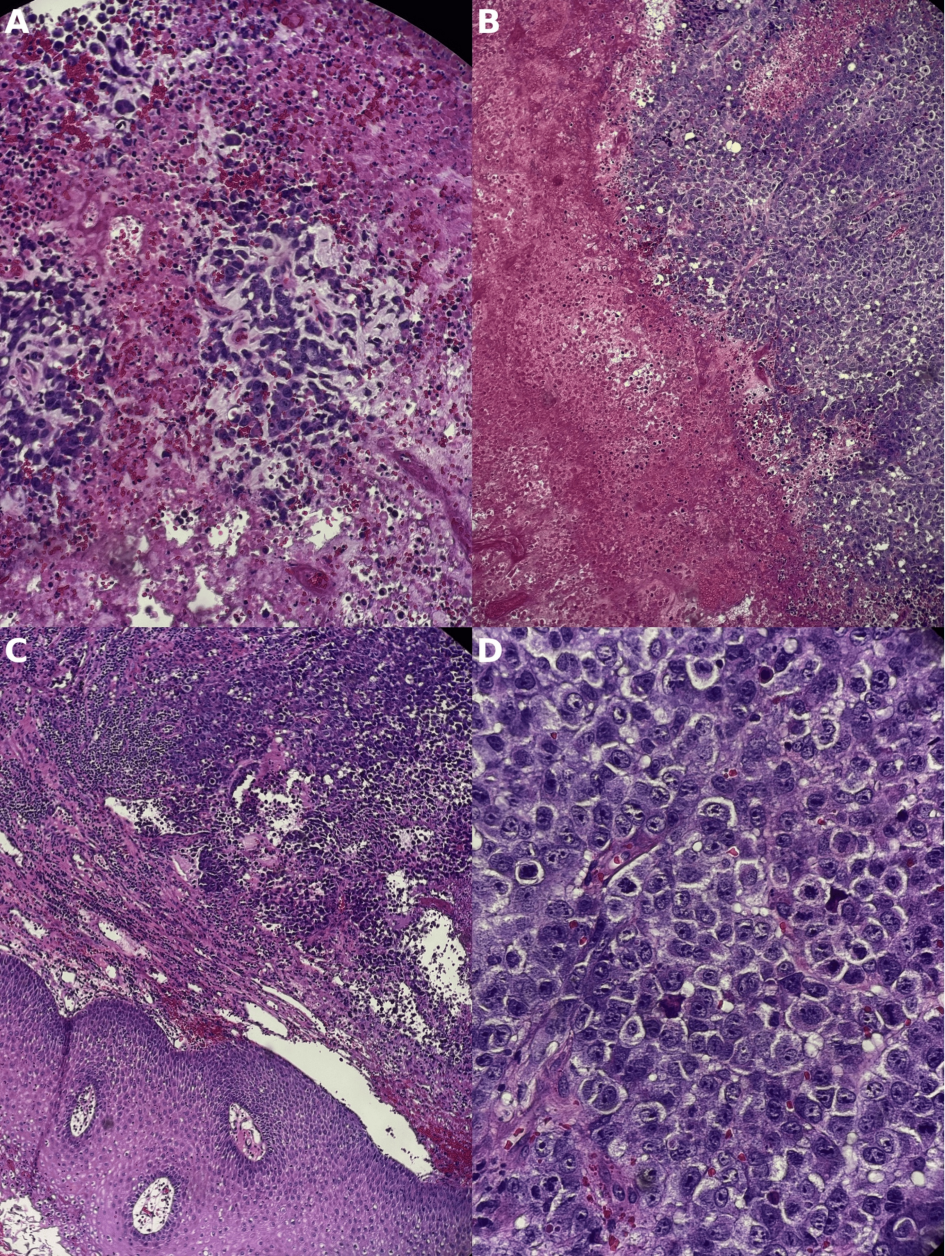
(A) Dyshesive tumor cells within myxoid matrix. (B) Broad areas of geographic necrosis are adjacent to the viable tumor. (C) Transition between normal oral mucosa and submucosal infiltrative tumor. (D) High-power image showing sheets of epithelioid tumor cells with vesicular nuclei and prominent nucleoli

Given the very rapid progression and low suspicion for an infectious etiology, a biopsy of the left tonsillar mass was performed and sent for outside consultation. Histopathological examination confirmed a diagnosis of SMARCA4-deficient undifferentiated malignant tumor. Immunohistochemistry demonstrated complete loss of BRG1 (also known as SMARCA4), strong CD34 and synaptophysin positivity, a mutant p53 null pattern, and faint focal epithelial membrane antigen (EMA) expression (Figure 1). This immunophenotype mirrored that described in thoracic SMARCA4-deficient tumors. Molecular profiling showed programmed death-ligand 1 (PD-L1) expression in the 5-10% range and an intermediate tumor mutational burden (TMB) of 8 mut/Mb.

**Figure 2:**
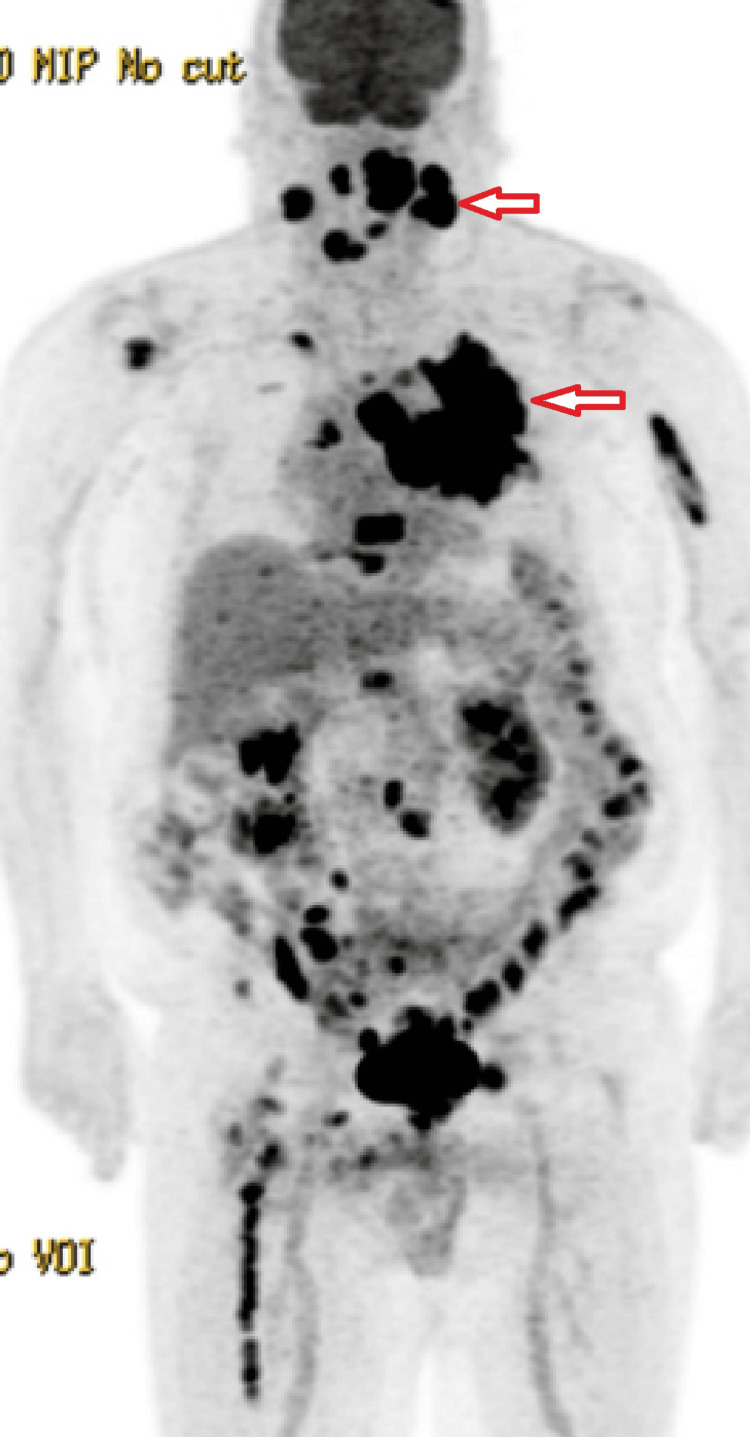
CT/PET at diagnosis with tonsillar mass and lung mass

Staging with CT/PET imaging revealed a markedly enlarged and hypermetabolic left tonsil consistent with a neoplastic process (Figure 2). There was bilateral level IIa lymphadenopathy with intense metabolic activity consistent with metastatic lymphadenopathy, a 6.5 × 5.6 cm hypermetabolic left upper lobe lung mass, and mediastinal lymphadenopathy. Additionally, multiple areas of increased metabolic activity were seen throughout the skeletal system, consistent with skeletal metastases.

Given the patient’s rapid clinical deterioration and inability to maintain oral intake, urgent systemic therapy was initiated with carboplatin, paclitaxel, and pembrolizumab while awaiting gastrostomy tube placement, which was completed four days later. Shortly after initiating therapy, the patient’s course was complicated by refeeding syndrome and appendicitis, which progressed to perforation and abscess formation, necessitating percutaneous biliary drain placement and antibiotic therapy. During this time, the left neck mass continued to grow, and he developed a new right-sided neck mass. Due to the presence of an active infection and an indwelling drain, he received single-agent pembrolizumab with cycle two of treatment.

**Figure 3:**
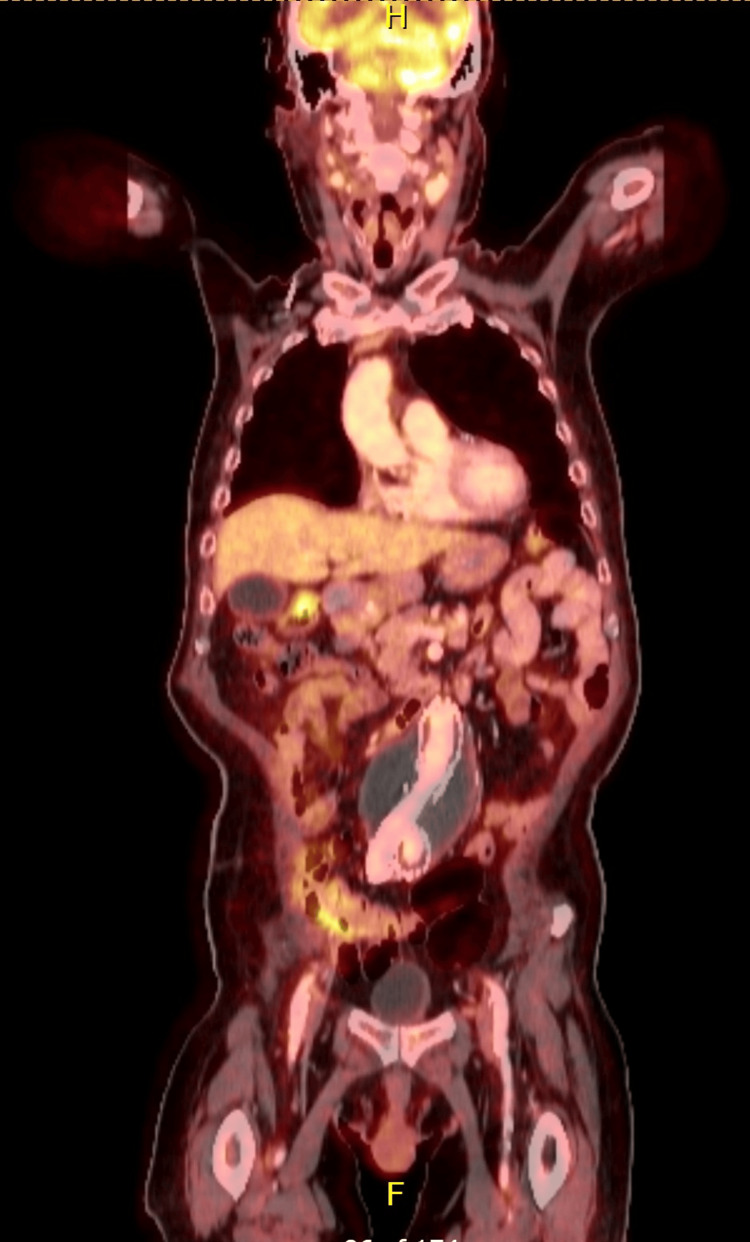
CT/PET with complete remission after treatment

After stabilization and resolution of the infection, the patient was discharged home after 23 days, wheelchair bound due to profound deconditioning. He resumed the full regimen of carboplatin, paclitaxel, and pembrolizumab every three weeks. His clinical course was further complicated by the development of a pulmonary embolism. Despite these setbacks, the bilateral neck masses began to shrink after three treatment cycles. A follow-up CT/PET scan after six cycles demonstrated a complete remission (Figure 3). The patient remains on maintenance pembrolizumab, and his functional status continues to improve.

## Discussion

SMARCA4-deficient undifferentiated tumors are newly characterized and poorly understood entities that exhibit highly aggressive clinical behavior. The diagnosis in this case was confirmed by loss of Brahma-related gene 1 (BRG1) expression and the characteristic immunohistochemical profile [3]. These tumors are most often reported in the thoracic cavity, with rare reports of extrapulmonary involvement. This case represents one of the few documented instances of a SMARCA4-deficient undifferentiated tumor arising in the head and neck region.

The clinical presentation was striking for its rapid progression and widespread metastatic involvement at the time of diagnosis. Early misdiagnosis as a benign infectious process, such as tonsillitis, is understandable, but it highlights the importance of maintaining a high index of suspicion for malignancy in elderly patients with rapidly evolving symptoms and systemic decline.

There are currently no standardized treatment protocols for SMARCA4-deficient undifferentiated tumors. Platinum-based chemotherapy in combination with immune checkpoint inhibitors has shown promise, particularly in tumors with PD-L1 expression or intermediate TMB [2,4]. In this case, despite severe complications including refeeding syndrome, appendicitis, infection, and pulmonary embolism, the patient achieved a complete response after six cycles of chemoimmunotherapy. This remission underscores the potential utility of this regimen in managing SMARCA4-deficient undifferentiated tumors.

## Conclusions

SMARCA4-deficient undifferentiated tumors are rare and highly aggressive, with most cases arising in the thoracic region. This case illustrates a rare presentation in the head and neck with widespread metastatic disease and highlights the potential for complete remission with a chemoimmunotherapy regimen of carboplatin, paclitaxel, and pembrolizumab. Early recognition and biopsy of atypical lesions, even those that mimic benign conditions, are critical for guiding timely diagnosis and treatment. Further research is needed to define optimal therapeutic strategies and improve outcomes for this devastating disease.

## References

[1] Pasricha S, Goyal S, Kamboj M (2024). Primary oropharyngeal SMARCA4-deficient carcinoma: expanding the diagnostic spectrum in head and neck cancer. Head Neck Pathol.

[2] Le Loarer F, Watson S, Pierron G (2015). SMARCA4 inactivation defines a group of undifferentiated thoracic malignancies transcriptionally related to BAF-deficient sarcomas. Nat Genet.

[3] Liu Z, Li N, Liu J (2025). SMARCA4-deficient undifferentiated thoracic tumor: a case report and literature review. J Thorac Dis.

[4] Shinno Y, Yoshida A, Masuda K (2022). Efficacy of immune checkpoint inhibitors in SMARCA4-deficient thoracic tumor. Clin Lung Cancer.

